# Distinct kinesiophobia profiles and associated factors in patients undergoing coronary artery bypass grafting

**DOI:** 10.3389/fpsyg.2026.1702153

**Published:** 2026-04-21

**Authors:** Qi Luo, Xiaojing Guo, Yi Xu, Yanqiu Yang, Mingzi Li

**Affiliations:** 1 Beijing Anzhen Hospital Affiliated to Capital Medical University, Beijing, China; 2 Peking University School of Nursing, Beijing, China

**Keywords:** coronary artery bypass grafting, determinants, exercise avoidance, Kinesiophobia, latent profile analysis

## Abstract

**Introduction:**

Kinesiophobia, or fear of movement, is common among patients undergoing coronary artery bypass grafting (CABG) and may hinder postoperative rehabilitation. However, little is known about its heterogeneity in this population. This study aimed to distinct kinesiophobia profiles and explore associated factors to inform targeted interventions.

**Methods:**

A cross-sectional survey was conducted among hospitalized CABG patients in Beijing, China, between October and November 2024. Kinesiophobia was assessed using the Tampa Scale for Kinesiophobia Heart Latent profile analysis (LPA) was applied to classify kinesiophobia subgroups. Chi-square tests and binary logistic regression were used to examine associations with sociodemographic variables, disease-related characteristics, social support, self-efficacy, and anxiety.

**Results:**

A total of 201 patients were included. LPA identified two subgroups: low kinesiophobia (46.8%) and high kinesiophobia (53.2%). Across both profiles, participants reported relatively low concern about “perceived danger for heart problems” but greater avoidance of exercise, indicating that exercise avoidance may represent a core characteristic of kinesiophobia in this population. Logistic regression further revealed that lower educational levels (middle school or below, OR = 3.59; high school/vocational, OR = 2.89) and higher anxiety levels (OR = 1.08) were significantly associated with membership in the high-kinesiophobia group.

**Discussion:**

Early identification of high-risk subgroups, particularly those with limited educational background or elevated anxiety, and the implementation of tailored psychological and educational interventions may help reduce kinesiophobia and promote postoperative recovery. Future longitudinal studies are needed to clarify its trajectory and to evaluate the effectiveness of targeted intervention strategies across perioperative and postoperative phases.

## 1 Introduction

According to the World Health Organization, cardiovascular diseases are the leading cause of global mortality, accounting for approximately 31% of all deaths worldwide (Wang et al., 2023). Among these, coronary heart disease (CHD) is a leading condition requiring cardiovascular surgical interventions and poses a significant threat to public health. While the incidence and mortality rates of CHD have declined in developed countries, they continue to rise in developing nations, placing a heavy burden on healthcare systems (Roth et al., 2020). Current treatment strategies for CHD include pharmacological therapy, interventional procedures, and surgical interventions (Feder and Griffiths, 1998; Mundth and Austen, 1975). Among these, coronary artery bypass grafting (CABG) is the preferred surgical option for complex coronary lesions and is widely recognized as one of the most effective surgical treatments for CHD (Doenst et al., 2019).

Exercise is a cornerstone of secondary prevention in cardiovascular disease, as it slows atherosclerotic progression, improves cardiopulmonary function, and reduces the risk of adverse cardiovascular events (Dibben et al., 2023). Despite these well-documented benefits, adherence to exercise regimens remains suboptimal. It has been reported that 50%–87% of patients who are able to exercise do not participate regularly (Bäck et al., 2016), and approximately 70% fail to meet recommended activity levels within the first year after treatment (Mons et al., 2014). This gap highlights the need to better understand the barriers to exercise participation in this population.

Kinesiophobia, or fear of movement, is a key psychological barrier to exercise. It is defined as an excessive, irrational, and debilitating fear of physical activity arising from a perceived vulnerability to injury or reinjury (Bäck et al., 2012). Grounded in the fear-avoidance model, individuals who catastrophize physical sensations may develop avoidance behaviors, leading to reduced activity, physical deconditioning, and adverse health outcomes (Gülşen et al., 2025). In the context of cardiovascular disease, patients may misinterpret normal physiological responses to exercise (e.g., increased heart rate or chest discomfort) as signs of cardiac danger, thereby reinforcing fear and avoidance (Zhang et al., 2025). Kinesiophobia is prevalent among patients with heart disease, with rates ranging from 39.20% to 82.57% following cardiac surgery, and is associated with functional decline and impaired postoperative recovery (Zeng et al., 2024).

Patients undergoing CABG may be particularly vulnerable to kinesiophobia due to surgical stress and heightened health concerns (Pedramrazi et al., 2024). However, the severity of kinesiophobia varies considerably across individuals and is likely influenced by a range of demographic, clinical, and psychological factors (Liu et al., 2024; Li et al., 2024). Such variability indicates that kinesiophobia is not a uniform construct but rather a heterogeneous phenomenon. Therefore, identifying distinct kinesiophobia subtypes and their associated factors is essential for informing targeted interventions and optimizing recovery outcomes in CABG patients.

Latent profile analysis (LPA) identifies potential characteristics of individuals based on their response patterns on specific measurement items, categorizing individuals with similar symptoms into different categories, which can better distinguish heterogeneity between individuals (Spurk et al., 2020). This approach allows for a better understanding of heterogeneity within populations. Despite the growing recognition of kinesiophobia as a significant barrier to postoperative rehabilitation, research on its heterogeneity in CABG candidates remains scarce. Understanding the distinct subgroups of kinesiophobia may provide insights into tailored intervention strategies. Therefore, the objectives of this study are: (1) to classify kinesiophobia subgroups in patients undergoing CABG using LPA; (2) to identify factors associated with kinesiophobia across different subgroups.

## 2 Materials and methods

### 2.1 Design

This observational, cross-sectional study aimed to identify distinct profiles of kinesiophobia and examine their associated factors among patients undergoing CABG. A total of 201 hospitalized patients undergoing CABG surgery were recruited from a tertiary general hospital in Beijing, China, between October and November 2024, and they completed the survey during their hospitalization. All participants completed the survey during the preoperative hospitalization period. Written informed consent was obtained from all participants prior to data collection.

### 2.2 Participants

Patients were recruited from the cardiology wards of a tertiary general hospital in Beijing, China, during the study period. The inclusion criteria were as follows: (1) Met the diagnostic criteria for CHD as outlined in the Guidelines for Primary Prevention of Cardiovascular Disease in China; (2) undergoing first-time CABG surgery with surgical indications; (3) Aged ≥18 years; (4) Classified as New York Heart Association (NYHA) functional class ≤III; (5) Provided informed consent to participate in the study.

The exclusion criteria were as follows: (1) Congenital motor dysfunction; (2) Lack of verbal communication abilities or presence of psychiatric disorders; (3) Unstable acute cardiovascular events; (4) Severe dysfunction or significant impairment of the respiratory system, liver, or kidneys.

### 2.3 Variables and measures

A self-designed questionnaire was used to collect general information, comprising two domains: sociodemographic and disease-related characteristics. The sociodemographic variables included age, sex, body mass index (BMI), educational level, and marital status. Disease-related variables encompassed disease duration and comorbid conditions.

Kinesiophobia was assessed using the Tampa Scale for Kinesiophobia Heart (TSK-SV Heart), the most widely validated tool for evaluating kinesiophobia in patients with heart disease (Bäck et al., 2012). It has been adapted into multiple languages, making it a globally recognized measure. This 17-item scale evaluates four key dimensions: two behavior-focused constructs, “Avoidance of exercise” and “Dysfunctional self,” which reflect irrational beliefs and avoidance behaviors related to rehabilitation; and two belief-oriented constructs, “Perceived danger for heart problem” and “Fear of injury,” which capture patients’ perceptions of risk and fear associated with exercise (Vlaeyen et al., 1995). Each item is rated on a 4-point Likert scale, ranging from strongly disagree to strongly agree, with total scores ranging from 17 to 68. Higher scores indicate greater levels of kinesiophobia.

Social support was assessed using the Social Support Rating Scale (SSRS), a widely used instrument originally developed by Xiao (1994). The scale consists of 10 items covering three dimensions: Objective Support, Subjective Support, and Utilization of Social Support. The total score is calculated as the sum of all items, with higher scores indicating greater levels of social support.

Self-efficacy in this study was measured using the Cardiac Exercise Self-Efficacy Instrument (CESEI), which was developed by Hickey et al., (1992) to measure exercise self-efficacy in patients undergoing cardiac rehabilitation, and translated and validated by Yuxiao et al. (2021). The Chinese version consists of 16 items rated on a 5-point Likert scale, ranging from 1 (very little confidence) to 5 (very strong confidence). The total score ranges from 16 to 80, with higher scores reflecting greater self-efficacy in engaging in exercise during rehabilitation.

Anxiety levels were evaluated using the Generalized Anxiety Disorder-7 (GAD-7), a brief and validated self-report measure (Spitzer et al., 2006). The GAD-7 consists of 7 items assessing the frequency of anxiety symptoms over the past 2 weeks. Each item is rated on a 4-point Likert scale: 0 (not at all), 1 (several days), 2 (more than half the days), and 3 (nearly every day). The total score ranges from 0 to 21, with higher scores indicating greater anxiety severity.

### 2.4 Statistical analysis

Statistical analyses were performed using SPSS version 26.0 (IBM Corp., Armonk, NY, USA) and LPA in Mplus version 8.3. Descriptive statistics, Chi-square tests, independent-samples *t*-test, and logistic regression analyses were performed in SPSS. Categorical variables were presented as frequencies and odds ratios, while continuous variables were summarized as means ± standard deviations (SDs).

LPA, a person-centered analytical approach, was employed to identify latent subgroups within the dataset by capturing individual heterogeneity based on continuous variables (Ferguson et al., 2020). Compared to traditional classification methods, LPA has demonstrated superior precision and more meaningful subgroup identification (Spurk et al., 2020). In this study, each dimension of the TSK-SV Heart scale was used as a key variable in constructing the latent profile model. To determine the best-fitting model, multiple fit indices and statistical criteria were evaluated. The Lo–Mendell–Rubin (LMR) test and the bootstrap likelihood ratio test (BLRT) were used to compare models with k and k − 1 classes, where a significant *p*-value supported the k-class model as a better fit. Model selection was further guided by entropy, the Akaike Information Criterion (AIC), the Bayesian Information Criterion (BIC), the adjusted Bayesian Information Criterion (aBIC), and the interpretability of the latent classes (Tein et al., 2013). Lower AIC, BIC, and aBIC values indicated improved model fit. Entropy values were used to assess the classification accuracy of each model, with higher values indicating more accurate categorization (ideally above 0.80). Additionally, average class membership probabilities were examined, with values of ≥0.80 deemed to indicate a stable and reliable classification solution. To ensure the statistical reliability, each latent profile was required to include at least 5% of the total sample, as smaller proportions may lead to unstable parameter estimates and unreliable conclusions.

Once the optimal latent profile model was identified, Chi-square tests and Fisher’s exact test were used to compare differences among the identified profiles. Univariate analyses were conducted to identify potential predictors for inclusion in subsequent multivariate analysis. To prevent the exclusion of potential important predictors, a *p*-value threshold of <0.25 was adopted during the initial variable selection phase (Hosmer et al., 2013). Subsequently, logistic regression was performed to assess the relationships between multiple factors and the latent profiles. Multicollinearity among independent variables was assessed using variance inflation factors to ensure no significant collinearity. To control for confounding effects, the backward likelihood ratio method was applied for variable selection and partial likelihood parameter estimation. Odds ratios (ORs) and 95% confidence intervals (CIs) were used to quantify the magnitude and direction of associations, with statistical significance set at *p* < 0.05.

## 3 Results

### 3.1 Characteristics of the sample

A total of 201 adults were included in this study. Participants’ ages ranged from 39 to 82 years, with a mean of 60.25 years (SD = 9.447). The majority were male (79.6%). In terms of education level, 54.2% had completed middle school or below. 94.0% of the participants were married, and 87.4% had at least one coexisting chronic disease. Detailed demographic and clinical characteristics are presented in Table 1.

**Table 1:** Participant characteristics.

Project (*n*, %)	Total (*N* = 201)	Class 1 (*n* = 94)	Class 2 (*n* = 107)	*x^2^*/*t*	*p*-value
Gender				0.004^a^	0.951
Male	160 (79.6)	75 (79.8)	85 (79.4)		
Female	41 (20.4)	19 (20.2)	22 (20.6)		
BMI				1.478^a^	0.224
18.5 ~ 24.9	85 (42.3)	44 (46.8)	41 (38.3)		
≥25	116 (57.7)	50 (53.2)	66 (61.7)		
Education				**13.061** ^**a**^	**0.001**
Middle school or below	109 (54.2)	42 (44.7)	67 (62.7)		
High school or vocational	48 (23.9)	21 (22.3)	27 (25.2)		
College or above	44 (21.9)	31 (32.9)	13 (12.1)		
Marital status				1.840^b^	0.431
Single	5 (2.5)	2 (2.1)	3 (2.8)		
Married	189 (94.0)	87 (92.6)	102 (95.3)		
Divorced/Widowed	7 (3.5)	5 (5.3)	2 (1.9)		
Disease duration				0.252^a^	0.882
<1 year	57 (28.4)	28 (29.8)	29 (27.1)		
1–5 years	89 (44.3)	40 (42.6)	49 (45.8)		
>5 years	55 (27.4)	26 (27.7)	29 (27.1)		
Combined with other chronic diseases				0.978^a^	0.323
No	25 (12.4)	14 (14.9)	11 (10.3)		
Yes	176 (87.6)	80 (85.1)	96 (89.7)		
Smoking status				0.355^a^	0.551
No	96 (47.8)	47 (50.0)	47 (45.8)		
Yes	105 (52.2)	49 (50.0)	58 (54.2)		
Alcohol consumption				0.057^a^	0.811
No	133 (66.2)	63 (67.0)	70 (65.4)		
Yes	68 (33.8)	31 (33.0)	37 (34.6)		
Social support	42.01 (6.173)	42.61 (6.229)	41.49 (6.103)	1.286^c^	0.200
Self-efficacy	**51.40 (9.967)**	**53.23 (9.337)**	**49.79 (10.247)**	**2.477** ^**c**^	**0.040**
Anxiety	11.04 (4.679)	10.37 (3.959)	11.63 (5.007)	−1.950^c^	0.053

a Chi-square tests.

b Fisher’s precision probability test.

c Independent-samples *t*-test.

Bold values indicate variables that showed statistically significant differences between the two kinesiophobia profiles in univariate analyses (Chi-square tests for categorical variables and independent-samples *t*-tests for continuous variables, *p* < 0.05).

### 3.2 Potential profiles of kinesiophobia

LPA was performed with one- to five-class solutions, with the corresponding fit indices presented in Table 2. Although fit indices (AIC, BIC, aBIC) improved with more classes, the two-class model was preferred: both the LMR (*p* = 0.008) and BLRT (*p* < 0.001) were significant, while the three-class model’s LMR was not (*p* = 0.276), indicating limited improvement with an additional class. Despite relatively low entropy (0.590), average class membership probabilities were high (87.7% and 88.2%; Table 3), supporting reliable classification. The two-class solution also offered clear interpretability and clinical relevance, distinguishing low- and high-kinesiophobia patients, and was therefore selected as optimal.

**Table 2:** Fit indices from latent profile analysis (*N* = 201).

Models	AIC	BIC	aBIC	Entropy	*p*-value		Class probability
					LMR	BLRT	
1	2918.375	2944.801	2919.456	–	–	–	–
**2**	**2848.651**	**2891.594**	**2850.408**	**0.590**	**0.0079**	**<0.0001**	**0.468/0.532**
3	2823.532	2882.991	2825.965	0.742	0.2757	<0.0001	0.318/0.647/0.035
4	2800.628	2876.604	2803.737	0.797	0.1603	<0.0001	0.015/0.5520.398/0.035
5	2796.198	2888.691	2799.983	0.830	0.3416	<0.1071	0.015/0.403/0.542/0.005/0.035

Bold values indicate the selected optimal model. The two-class solution was chosen based on significant LMR and BLRT results (*p* < 0.05), while the non-significant LMR for the three-class model indicated no substantial improvement with additional classes.

**Table 3:** Probabilities of belonging to two latent classes.

Latent category	C1	C2
C1	0.877	0.123
C2	0.118	0.882

**Figure 1:**
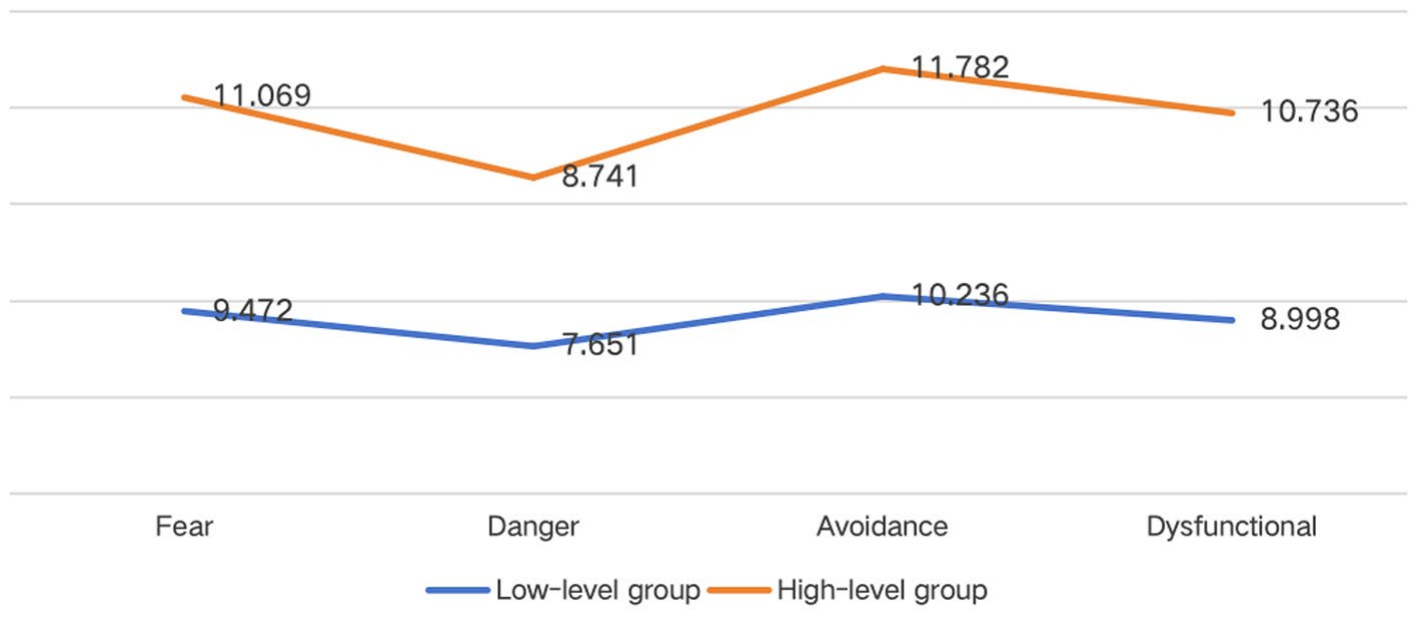
Kinesiophobia scores across two latent profiles in patients undergoing coronary artery bypass grafting. Fear, fear of injury; danger, perceived danger for heart problem; avoidance: avoidance of exercise; dysfunctional, dysfunctional self.

The LPA findings identified two distinct kinesiophobia profiles among patients undergoing CABG (Figure 1). Based on the conditional means of scale dimensions within each class, Class 1 (*n* = 94, 46.8%) was categorized as the low-level kinesiophobia group (C1), while Class 2 (*n* = 107, 53.2%) was identified as the high-level kinesiophobia group (C2). As illustrated in Figure 1, both groups demonstrated relatively low scores in the “Perceived danger for heart problem” dimension, whereas the “Avoidance of exercise” dimension showed consistently higher scores in both groups.

### 3.3 Factors related to potential profiles of kinesiophobia

The Chi-square tests revealed a significant association between the two kinesiophobia profiles and education level (*x*^2^ = 13.061, *p* = 0.001), while the independent-samples t-test identified a significant difference in self-efficacy between the two profiles (*t* = 2.477, *p* = 0.040; Table 1). To explore potential predictors of profile membership, binary logistic regression was performed, using the low-level kinesiophobia group as the reference category. Variables with *p* < 0.25 in the univariate analysis were included in the model.

Compared to patients with a college degree or higher, those with middle school or below (OR: 3.585, CI: 1.544–8.324) and high school or vocational education (OR: 2.889, CI: 1.153–7.236) had significantly higher odds of belonging to the high-level kinesiophobia group. And as expected, higher anxiety levels were associated with an increased likelihood of classification into the high-level kinesiophobia group rather than the low-level group (OR = 1.075, 95% CI = 1.001–1.154) (see Table 4).

**Table 4:** Binary logistic regression results predicting profile membership.

Project	*β*	SE	Wald χ^2^	*p*	OR	95%CI
BMI						
18.5 ~ 24.9	−0.615	0.316	3.788	0.052	0.541	0.291–1.044
≥25	–	–	–	–	–	–
Education						
Middle school or below	**1.277**	**0.43**	**8.824**	**0.003**	**3.585**	**1.544–8.324**
High school or vocational	**1.061**	**0.468**	**5.127**	**0.024**	**2.889**	**1.153–7.236**
College or above	–	–	–	–	–	–
Social support	−0.015	0.026	0.328	0.567	0.985	0.936–1.037
Self-efficacy	−0.031	0.017	3.278	0.07	0.97	0.938–1.003
Anxiety	**0.073**	**0.036**	**4.001**	**0.045**	**1.075**	**1.001–1.154**

Bold values indicate variables that were significantly associated with profile membership in the binary logistic regression (*p* < 0.05).

## 4 Discussion

This study aimed to identify potential kinesiophobia profiles among patients undergoing CABG and examine their associated factors. Two distinct kinesiophobia subgroups were identified: a low-level kinesiophobia group (46.8%) and a high-level kinesiophobia group (53.2%). Lower education level and higher anxiety were identified as key factors associated with high-level kinesiophobia, underscoring the crucial role of psychological and cognitive factors in shaping patients’ perceptions and behaviors towards exercise.

Compared to previous studies, our findings provide a more nuanced understanding of kinesiophobia in cardiac patients. Our results are largely congruent with existing literature; for instance, Wang et al. (2023) identified three distinct kinesiophobia subgroups (low, moderate, and high) among patients with CHD, with 23.8% of the population categorized into the high-kinesiophobia group. Similarly, a prospective cohort study by Keessen et al. (2022a) reported that approximately 22.8% of cardiac patients exhibited moderate-to-severe kinesiophobia at the time of hospital discharge. While these prevalence rates establish a critical baseline, our research specifically targeted patients undergoing CABG. These individuals often face greater disease severity, and heightened surgical anxiety, all of which may contribute to more pronounced or complex kinesiophobia profiles than those observed in general CHD populations.

Furthermore, the clinical importance of identifying these high-kinesiophobia latent profiles is underscored by the prognostic impact of kinesiophobia on recovery. As demonstrated by Keessen et al. (2022a) in their longitudinal analysis, kinesiophobia serves as a substantial barrier to secondary prevention, where each one-point increase in the TSK score is associated with an 8% decrease in the likelihood of initiating cardiac rehabilitation. By identifying specific CABG patient subtypes at higher risk of movement-related fear, our study provides a foundation for targeted behavioral interventions aimed at mitigating this fear and, consequently, improving the uptake of essential rehabilitation services.

Additionally, our study employed a dimension-based LPA approach across four kinesiophobia domains, enabling a structured evaluation of its core components. Both groups showed low scores in “Perceived danger for heart problems” but high scores in “Avoidance of exercise,” indicating that fear-driven avoidance, rather than perceived physical risk, is the primary barrier to exercise. Although patients undergoing CABG may receive medical reassurance, prolonged inactivity, deconditioning, and surgical anxiety can maintain excessive avoidance, reinforcing a cycle of fear and inactivity. These findings highlight the need for interventions that specifically address maladaptive avoidance behaviors rather than focusing solely on risk perception.

Regarding the key factors influencing kinesiophobia, our study identified lower education levels and elevated anxiety as key factors associated with higher kinesiophobia in CABG patients. Lower education likely limits health literacy and understanding of the benefits and safety of post-surgical exercise, reinforcing fear-driven avoidance. This is consistent with previous meta-analysis evidence highlighting education as a major determinant of kinesiophobia (Liu et al., 2024). Moreover, a Cochrane systematic review highlights that structured, multifaceted interventions—particularly those incorporating targeted communication and patient education—effectively enhance engagement with and adherence to cardiac rehabilitation programs (Long et al., 2026). High anxiety also contributes to severe kinesiophobia, as patients may catastrophize normal cardiac sensations and perceive them as signs of graft failure, consistent with the fear-avoidance model (Alpalhão et al., 2022). Interventions that enhance self-efficacy and provide timely psychological support—such as remote coaching programs—have been shown to prevent the cycle of fear, inactivity, and deconditioning, and may be particularly beneficial for patients identified in the high-kinesiophobia profile (Keessen et al., 2022b). These findings suggest that early, tailored education and behavioral interventions are critical to mitigating kinesiophobia and supporting successful postoperative recovery.

From a clinical perspective, these findings highlight the need for tailored interventions to address kinesiophobia in patients undergoing CABG. Given that kinesiophobia can negatively impact postoperative recovery and long-term cardiac rehabilitation, early identification and intervention during the preoperative hospital stay are essential. Healthcare providers should integrate educational strategies to enhance patients’ understanding of the importance, safety and benefits of postoperative exercise, particularly among those with lower education levels. In addition to education, Cognitive-behavioral interventions aimed at reducing anxiety and modifying maladaptive fear-related beliefs may play a crucial role in alleviating kinesiophobia. Moreover, integrating psychological assessments into preoperative evaluations could facilitate the early detection of high-risk individuals, personalized interventions. By implementing such preemptive strategies during hospitalization, healthcare teams can not only support smoother postoperative rehabilitation but also foster sustained engagement in cardiac exercise programs after discharge, ultimately enhancing long-term cardiovascular outcomes.

Despite its contributions, this study has several limitations. First, the relatively small sample size and single-center design may limit the generalizability of the findings. Future research should consider multi-center studies with larger, more diverse samples to validate the identified kinesiophobia profiles. Second, the cross-sectional design precludes causal inferences regarding the relationships between kinesiophobia, education, and anxiety. Longitudinal studies are needed to explore how kinesiophobia evolves over time and whether interventions can effectively modify these profiles. Lastly, the reliance on self-reported measures may introduce response biases; incorporating objective measures of exercise and psychological distress could enhance the robustness of future investigations.

## 5 Conclusion

In conclusion, this study identified two distinct kinesiophobia profiles among patients undergoing CABG and highlighted the significant roles of education level and anxiety in shaping these fear-related avoidance behaviors. These findings emphasize the necessity of integrating psychological and educational interventions into cardiac rehabilitation programs to reduce kinesiophobia and enhance postoperative recovery. Future research should further investigate the longitudinal trajectory of kinesiophobia throughout the perioperative period and postoperative cardiac rehabilitation, as well as develop targeted interventions to effectively address this issue and optimize long-term recovery outcomes.

## Data Availability

The raw data supporting the conclusions of this article will be made available by the authors, without undue reservation.
